# MassSorter: a tool for administrating and analyzing data from mass spectrometry experiments on proteins with known amino acid sequences

**DOI:** 10.1186/1471-2105-7-42

**Published:** 2006-01-26

**Authors:** Harald Barsnes, Svein-Ole Mikalsen, Ingvar Eidhammer

**Affiliations:** 1 Department of informatics, University of Bergen, PB. 7800, N-5020 Bergen, Norway; 2 Computational Biology Unit, Bergen Center for Computational Science, UNIFOB/UIB, Thormoehlensgt. 55, N-5008 Bergen, Norway; 3 Institute for Cancer Research, Rikshospitalet-Radiumhospitalet University Hospital, Montebello, N-0310 Oslo, Norway

## Abstract

**Background:**

Proteomics is the study of the proteome, and is critical to the understanding of cellular processes. Two central and related tasks of proteomics are protein identification and protein characterization. Many small laboratories are interested in the characterization of a small number of proteins, e.g., how posttranslational modifications change under different conditions.

**Results:**

We have developed a software tool called MassSorter for administrating and analyzing data from peptide mass fingerprinting experiments on proteins with known amino acid sequences. It is meant for small scale mass spectrometry laboratories that are interested in posttranslational modifications of known proteins. Several experiments can be compared simultaneously, and the matched and unmatched peak values are clearly indicated. The hits can be sorted according to m/z values (default) or according to the sequence of the protein. Filters defined by the user can mark autolytic protease peaks and other contaminating peaks (keratins, proteins co-migrating with the protein of interest, etc.). Unmatched peaks can be further analyzed for unexpected modifications by searches against a local version of the UniMod database. They can also be analyzed for unexpected cleavages, a highly useful feature for proteins that undergo maturation by proteolytic cleavage, creating new N- or C-terminals. Additional tools exist for visualization of the results, like sequence coverage, accuracy plots, different types of statistics, 3D models, etc. The program and a tutorial are freely available for academic users at .

**Conclusion:**

MassSorter has a number of useful features that can promote the analysis and administration of MS-data.

## Background

Proteomics is the study of the proteome, the protein complement of the genome, and is critical for the understanding of cellular biological processes. Two central and related tasks of proteomics are protein identification and protein characterization. Identification is commonly done by peptide mass fingerprinting (PMF), where the masses of a set of peptides from the protein(s) are determined by mass spectrometry (MS), followed by a search in a sequence database. Alternatively, single peptides can after MS be selected for fragmentation followed by another MS experiment (tandem MS or MS/MS). The resulting MS/MS spectra are used for searching in a sequence database. For reviews of mass spectrometry and bioinformatics in proteomics, see for example [1-4].

Although there has been an enormous increase in large-scale proteomics, there is still a need for tools for researchers concentrating on the characterization of single or a small number of proteins. One of the most important tasks for characterization of a known protein (known sequence) is the determination of posttranslational modifications, which can be done both by MS- and MS/MS experiments. Typically an MS experiment can discover that a modification has occurred, but not the position in the peptide (if there are several alternatives). The exact position can be determined by MS/MS.

There are a number of programs intended for the identification of proteins by PMF, e.g., MS-Fit, a program in ProteinProspector [5], Mascot [6], Profound [7], Aldente [8], Phenyx [9], GPMAW [10], etc. As a part of the search parameters, the user can choose different modifications believed to be present in the proteins analyzed, but other modifications are not considered. Thereby a partial characterization is also achieved. A program like MS-Screener [11] may promote the identification of proteins by removing common contaminating peaks from different samples. Other programs are directed towards further characterization of PMF data from the identified proteins. For example FindMod [12] can suggest modifications present in peptides, and FindPept [13] can suggest whether unexpected cleavages have occurred.

Among all these programs, only Phenyx has an administrative unit that collects and analyzes data from several experiments. Phenyx is mostly directed toward protein identification, and not the detailed and repeated analysis of single proteins. Furthermore, it is intended for large-scale, high-throughput MS and MS/MS, and it is machine-demanding. A software application for small-scale proteomics should include an administrative unit that can function as a database of results, and it should be possible to directly compare several experiments in a table or a spreadsheet. The analytical tools should be integrated around this administrative unit. All the analytical tools should have a uniform and user-friendly style, such that the data flow between the tools becomes easy. Furthermore, it should be platform and server independent, and optimized for small scale analysis.

We have developed a set of tools, MassSorter, that satisfies these requirements. MassSorter sorts, systematizes and analyzes data obtained from MS experiments where a known protein is analyzed for sequence coverage, posttranslational modifications, modifications occurring during sample handling, and induced chemical modifications. MassSorter functions as a database for all the peptides detected in the experiments and at the same time sorts the data according to given parameters by comparing obtained data with theoretical data. Data not recognized by this first comparison can go through a second round of analysis where other tools can suggest the origin of the still unidentified data. MassSorter is intended as a tool for small mass spectrometry laboratories that are interested in characterization of known proteins.

## Implementation

**Figure 1:**
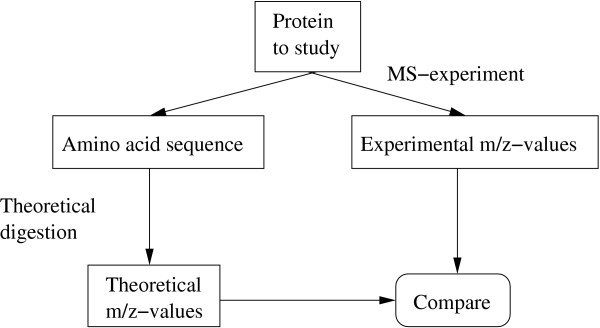
The basic procedure of MassSorter The experimental masses are compared to theoretical values.

The basic idea is to compare experimental m/z values from MS experiments with theoretical m/z values from a theoretical digestion of the same protein, as shown in Figure 1. The goal is to maximize the number of experimental values for which a possible origin can be found.

### Modifications, enzymes, terminals and amino acid data

A given set of default modifications, enzymes and terminals are included in MassSorter, along with information about the 20 standard amino acids. The user may add his/her own alternatives and/or edit the existing ones. In this way, MassSorter can be tailored to the individual user's needs. For a user-specified modification, the following data are needed: i) an abbreviation, ii) the amino acid(s) affected, iii) the mass modification, iv) a comment explaining the details of the modification, and v) a set of rules to determine where the modification can occur. For enzymes, a name and the cleavage rules must be specified. The user can also specify his/her own amino acids, as well as N- and C-terminals.

### Data tables

MassSorter contains three main data tables.

#### Theoretical background table (TBT)

The TBT contains the theoretical peptide peak list of the protein digested by a specific protease. It also contains the parameters used for theoretical digestion, including considered modifications.

#### The experimental data table (EDT)

An EDT contains four main features for each experiment: i) information about the experiment (protein, enzyme and date), ii) experimental comments, iii) a list of expected posttranslational modifications, and iv) the peak list. Additional information about each peak (e.g., intensity and manually added comments) is also included. During the import of the peak list, it is possible to manually edit the data, e.g., remove a peak that the user identifies as noise, or add a peak that the instrumental processing software has not recognized.

#### The data sheet table (DST)

After importing the experimental peak lists into MassSorter, each list is compared to the theoretical peak list in the TBT. Each peptide mass from an experimental peak list is compared to all the theoretical peptide masses, and matches within the selected accuracy limit are detected. If an experimental peptide mass is unmatched, i.e. there is no matching theoretical peptide mass, the given peptide mass is compared to the peptide masses from the other MS experiments, if any. The results from all of these comparisons are visualized in a spreadsheet called the data sheet table (DST).

All the experimental masses for each MS experiment will appear in the DST. The comparison of experimental masses to theoretical masses may result in one of the three following color-coded alternatives in the DST:

1. A *primary match *between an experimental and a theoretical peptide mass, colored light green. Optionally, shadings of green can be used to indicate the peak intensity.

2. A *secondary match *between an experimental and a theoretical peptide mass, colored dark green.

3. An unmatched experimental peptide mass, colored yellow.

**Figure 2:**
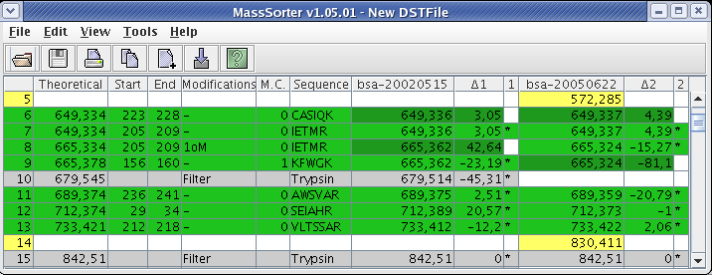
A fraction of a DST table for two MS experiments. Rows 11, 12, and 13 contain primary hits. Rows 6–7 and 8–9 are examples of peaks with secondary hits. Rows 5 and 14 contain unmatched masses, and rows 10 and 15 correspond to filter peaks (from a trypsin sample).

Figure 2 shows a fraction of a DST. The mentioned colors are the default settings, but they can be changed by the user.

The terms primary and secondary matches are used to separate two types of matches between an experimental and a theoretical peptide mass. When comparing an experimental peptide mass to the theoretical peptide masses, more than one theoretical peptide may match within the given accuracy limit. Without further analysis of the alternatives, it is not possible to determine which is the correct match.

Although the closest match might be the correct match, this is not always the case. It is even possible that all the found matches are correct. The user of the program must therefore decide which of the matches to keep and use in the subsequent analysis. The accepted matches are labeled primary matches and the others are labeled secondary matches. As a default, the match with the least distance in ppm (part per million) or Dalton is labeled primary match and the others are labeled secondary matches.

When the comparison of an experimental mass and a theoretical mass results in a match, the theoretical peptide data are inserted into the DST on the same row as the experimental mass. The first six columns of the DST are therefore reserved for data about the theoretical peptides: the theoretical peptide mass, the start and end position of the peptide, added modifications, number of missed cleavages and the peptide sequence. For the unmatched masses, these columns are left empty. The following columns contain the information about the experiments, three columns per experiment.

There are two kinds of theoretical peptides; modified and unmodified. If a match with a modified theoretical peptide occurs, all the modifications have to be *possible *on the experimental peptide for the match to be allowed.

The analytical tools included in MassSorter are centrered around the DST. Several experiments can be displayed in one DST, and the analytical tools process all experiments shown in the DST.

### Detecting additional matches

After creating the initial version of the DST, there will probably still be a set of unmatched experimental masses. There are different reasons for this and MassSorter includes several tools for increasing the number of matches.

1. Masses belonging to additional proteins in the mixture, for example the protease used for digestion. There could also be contaminating proteins, like keratin or proteins comigrating with the protein of interest during gel electrophoresis. It is possible to use such proteins as *filters*, which are combined with the TBT. Filters can be defined in three different ways:

• As experimental masses, for example the autolytic peptide peaks from the protease

• As a protein sequence, which then has to be theoretically digested

• As masses defined by the user, either directly (the mass), or indirectly as a peptide sequence, from which the mass has to be calculated

2. Masses from peptides resulting from unexpected cleavage sites. This is handled by the tool *SequenceSuggester*, which compares an unmatched mass to any linear amino acid sequence (with and without given modifications) occurring in the protein.

3. Masses resulting from unexpected modifications. This is handled by the tool *UniModSearch *which searches a local version of the UniMod database [14,15] for unexpected modifications, or by the tool *ChangeModifications *which searches for other user-specified modifications.

The two last tools take an unmatched peptide mass as input and return a list of possible explanations. It is up to the user to accept or discard the suggested explanation(s). If an explanation is accepted, the match is inserted into the DST and is colored blue.

### Theoretical digestion

*ProteinDigester *is the tool MassSorter uses for the theoretical digestion. The basic input is an amino acid sequence along with a set of parameters that characterizes the digestion and the resulting mass calculation: i) the enzyme, ii) the peptide N- and C-terminals, iii) possible charges, iv) a list of possible modifications, v) limits for the properties of the peptides created (the minimum peptide mass, the maximum peptide mass, the minimum peptide length, the maximum number of missed cleavages), and vi) use of monoisotopic or average amino acid residue masses.

There already exist several programs for theoretical digestion, either as separate programs or as part of database search programs, see for example MS-Digest, a tool in ProteinProspector [5,16], and PeptideCutter [17]. However, we decided to develop our own to make the connection to the other tools faster and simpler.

### Presentation

The results are presented and viewed in several ways.

#### DST

The Data Sheet Table is displayed in a spreadsheet (Figure 2).

#### Report

*Report *is a tool on top of the DST where all the matched and unmatched masses per experiment are grouped and counted. In addition, the sequence coverage is calculated and visualized.

The experimental masses can be grouped according to the following matching types: i) matches with unmodified theoretical peptides, ii) matches with modified theoretical peptides, iii) unexpected matches, iv) matches with filter(s), v) unmatched masses, and vi) secondary matches. The number of experimental peptide masses and the resulting match percentage are also shown.

The second part of the Report visualizes the sequence coverage, both per experiment and combined for all experiments. The entire amino acid sequence is shown and the detected parts of the sequence are colored. The possible cleavage sites of the enzyme used are marked, and also the modified residues. The Report is connected to the DST, which means that if the user wants to know which of the detected peptides include a certain residue in the protein, the user can right click on the residue and a window will appear containing the peptide information from the DST. The Report also includes an option to view the amino acid sequence as a 3D model.

#### Protein Viewer – 3D modeling

**Figure 3:**
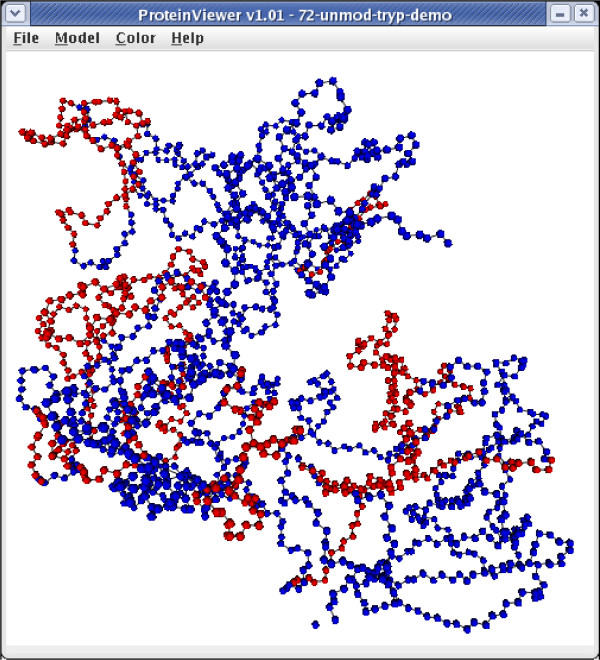
Screenshot of a protein structure (MMP2, ProteinDataBank 1CK7), where the covered residues are colored red, and the uncovered residues are colored blue.

*Protein Viewer *is a tool which creates a 3D model of the amino acid sequence of a protein. The input to the Protein Viewer is a PDB file [18], containing the structure data for the given protein. By combining the data in the PDB file with the data from the DST, it becomes possible to display a 3D model of the protein where the detected parts of the protein structure have one color and the undetected parts have another color. This makes it possible to determine which parts of the protein structure are covered, see Figure 3. The user can rotate, zoom and move the 3D model to get the wanted view of the protein structure. It is possible to right click on an atom to get details about the atom along with information about the peptides containing the selected residue. The user may also highlight certain modifications (e.g., all phosphorylated serines), specific positions in the protein (e.g., position 110, or 110–115), or certain amino acids (e.g., all tyrosines). Protein Viewer is an extension of a program written as part of a master thesis [19] and is based on a program written at University of California [20].

### Statistical analysis

After using the MassSorter tools for locating the origin of the unmatched masses, a complete sequence coverage is still unlikely to have been obtained. Our experience shows that a sequence coverage of 20–40% might be realistic in single experiments. Even if all the experimental peptide masses in an MS experiment are matched to theoretical peptide masses, a sequence coverage of 100% is still unlikely. The properties that may effect the sequence coverage can be divided into three categories: i) peptide properties, ii) instrument properties, and iii) protocol properties. Several papers have analyzed such properties, see for example [21-23]. The current version of MassSorter includes a tool, *PeptideStatistics*, that considers and makes statistics for the following peptide properties: hydropathy, peptide length, amino acid frequencies, cleavage site frequency and peptide mass. It also includes a tool, *Accuracy Statistics*, for statistical analysis of the spread of the matching accuracy to see if there are accuracy values that differ substantially from the rest. Analysing the details of the spread of the ppm (or Dalton) values might be a way of eliminating some of the less likely matches. This is because after calibration the spectrum is transformed into a spectrum where the peptide's accuracy values have a close to uniform spread. It is assumed that the correct matches will lie within a certain area of the range of accepted accuracy values. If there are any matches with a ppm (or Da) value far from the common range, this might be an indicator of an incorrect match. MassSorter therefore contains a tool for plotting the experimental m/z values against the accuracy values. Another way of detecting correct matches is to perform MS/MS experiments on the peptide mass in question and compare the results to the sequence of the proposed matching theoretical peptide.

*Fractional masses *(also known as the half decimal point rule [11]) is dependent on the ratio of the different atoms in the peptides. As several types of modifications will change this ratio, fractional mass may help to predict peptides with such modifications. Therefore, MassSorter can display a plot of fractional masses for the peaks obtained in the experiment. The constants and borders (four standard deviations) are calculated as in [24].

### User interface

The user interface was developed following the eight golden rules given in [25]. This has resulted in a uniform and easy to use interface, which can also easily be changed to accommodate specific user preferences.

### Diagram of MassSorter

**Figure 4:**
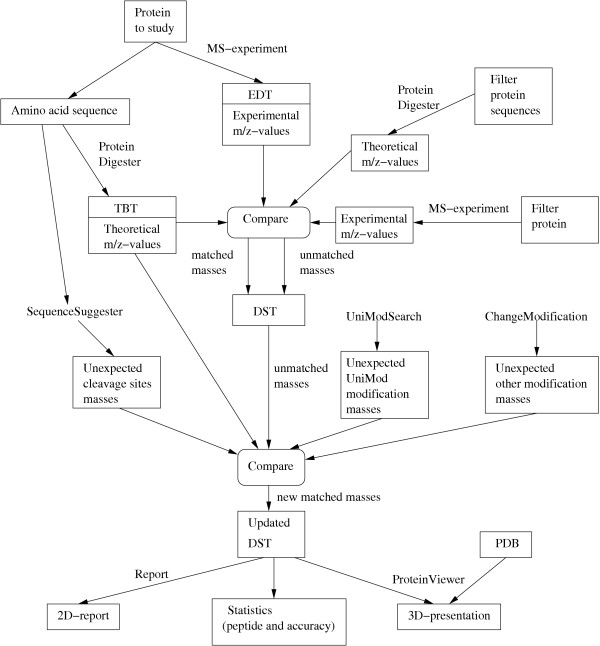
The basic tools and processes of MassSorter.

An overview of the basic tools and processes of MassSorter is shown in Figure 4.

## Results and discussion

**Figure 5:**
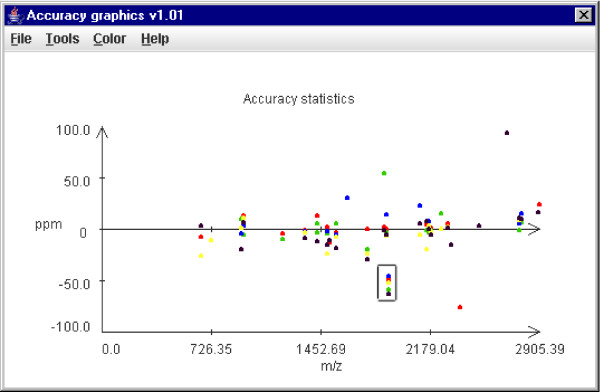
Screenshot of an accuracy plot for five PMF experiments of a protein. A peptide that consistently showed aberrant accuracies in the range of -40 to -60 ppm is boxed. Despite the hit within the accepted accuracy limit, the aberrant behavior strongly indicates that this is not the suggested peptide.

We have developed a software tool, MassSorter, for the administration and analysis of PMF experiments on proteins with known amino acid sequences. The software makes it possible to manually edit the experimental data if needed, to compare the detected peaks with a theoretical (in silico) digestion of the protein, to show several experiments in one spreadsheet, to easily visualize the similarities (e.g., the reproducibility of repeated experiments) and differences (e.g., new peptides showing up under certain conditions) between experiments, and to further analyze peaks that do not match any theoretical masses. Examples of cases where MassSorter promoted our analyses of PMF (MALDI-TOF-MS) spectra follow:

1. MMP-2 is a 72 kDa protease that may autoactivate by cleavage into a 62 kDa form. Both forms were studied by MALDI-TOF-MS [26]. The tryptic peak lists were imported into MassSorter and the two MMP-2 forms were assembled into one DST. The standard sorting of the data in the DST is according to increasing m/z ratio, but the data can also be sorted according to the amino acid sequence of the protein. This made it immediately evident that the 62 kDa form lacked all peaks N-terminal to position 115 (numbering according to GenBank accession number NP_004521). A peak at m/z 843.4 was present from the 62 kD form, but absent from the 72 kDa form. SequenceSuggester gave several alternatives for this m/z value, but only one contained a C-terminal arginine (or lysine). This peptide corresponded to positions 110–115. This is likely to be the correct N-terminus of the 62 kDa form [27]. This example is included in the tutorial.

2. It is considered an advantage to perform internal calibration in PMF experiments. If possible, autolysis peaks from the protease are used. Chymotrypsin is rarely used for identification purposes, but can be useful for characterization purposes. Bovine chymotrypsin (Sigma C-6423) gave a number of autolysis peaks that fitted with chymotryptic autodigestion. However, the most intense peak at m/z 1523.8 did not fit with standard rules of chymotryptic cleavage (C-terminal to F, Y, W, L). SequenceSuggester gave three alternative peptides within the 50 ppm accuracy limit. The results of subsequent experiments (tryptic cleavage of the chymotryptic peptide, C-terminal sequencing with carboxypeptidase Y, and partial post-source decay sequencing) were consistent with the one of the three suggested peptides, 149-ANTPDRLQQASLPL-162 (cleaved C-terminal to an N, with m/z 1523.8182). As a result, this peptide could be used as an internal calibrant in later experiments.

3. A protein was purified from cultured cells and tryptic peptides were analyzed by MALDI-TOF-MS. While most of the peptides showed a distribution of accuracies more or less around the theoretical values, one peptide consistently showed accuracies around -30 to -60 ppm, as shown in Figure 5. Its aberrant behavior was very obvious in the accuracy plot. If the peptide is indeed generated from this protein, it is unlikely to be the indicated peptide, but rather another peptide that contains an unknown modification. No reasonable suggestions were obtained by using UniModSearch. We therefore excluded this particular peptide from subsequent analyses.

MALDI-TOF-MS instruments are becoming more abundant, and they are very useful for both identification and characterization purposes. Many laboratories are interested in detailed characterizations of a low number of proteins. Such characterizations can be performed by manual analysis of the spectra and peak lists, but this is very time consuming. A simple and basic question valid both for identification and characterization purposes is "how reproducible are our results?". In a manual analysis, this would need lengthy comparisons of the peak lists. With MassSorter, the alignment of multiple experiments can be done in seconds after the import of the experimental data, and their reproducibilities and accuracies can be viewed in several ways. Furthermore, reproducible peaks that do not fit with the theoretical digestion of the protein, are immediately evident. Such peaks can be further analyzed by the two tools, SequenceSuggester and UniModSearch. The suggestions from these tools may be used as basis for subsequent experiments as indicated in example 2 above. If the structure of the protein is known and available as a PDB file, the detected peptides can be indicated in the 3D model of the protein. Specific peptides or amino acids can be highlighted in this model and this can be used for further interpretation of the results. For example a phosphorylation or glycosylation is unlikely to be hidden in the internal structures of a protein. There is also the possibility to manually add modification definitions, e.g., modifications not available in the UniMod database. It is therefore also easy to reanalyze old data if the user becomes aware of a modification that has not been considered before.

## Conclusion

We have developed MassSorter as a software tool for small-scale mass spectrometry laboratories performing PMF experiments. It is independent of the producer of the instruments and only needs the peak lists to be exported (or copied) as a text format. It has a number of features that facilitates administration and analysis of the data.

## Availability and requirements

• Project name: MassSorter

• Project home page: 

• Operating system(s): Platform independent

• Programming language: Java, Netbeans [28] used for the user interface

• Other requirements: Java 5.0 or higher, Java 3D 1.3.1

• License: The program is freely available for academic users after registration at the project's home page

## Authors' contributions

HB did the programming, contributed ideas and participated in writing the manuscript. SOM made the initial descriptions of the program, performed all mass spectrometry experiments, and participated in writing the manuscript. IE supervised the programming work, contributed ideas, and participated in writing the manuscript.
